# Anniversary issue of JCHIMP

**DOI:** 10.3402/jchimp.v1i4.15885

**Published:** 2012-01-26

**Authors:** Robert P. Ferguson

This is the fourth quarterly publication of the *Journal of Community Hospital Internal Medicine Perspectives* (JCHIMP), www.JCHIMP.org. It could be looked at as the journal's first birthday. It has the largest number of published manuscripts to date. The theme of this issue is case reports; it includes a perspective on case reports as well as numerous interesting cases that have been submitted by residents and faculty.

Publishing case reports is part of the journal's mission. JCHIMP was organized in 2010 to respond to various needs expressed in the internal medicine educational community, including the need for a journal with community hospital orientation that would be a scholarly vehicle for residents and faculty. After contracting with the publisher Co-Action Publishing based in Stockholm, Sweden, in 2010 and developing a mentoring relationship with David Solomon, editor of *Medical Education Online*, we first called for papers 1 year ago in January 2011. Initially, we limited our manuscript call to original research, perspective pieces, case reports, medical education innovations, and an electrocardiogram column. In this, issue number 4, we have expanded to include brief reports and medical imaging. In our next issue, we expect to inaugurate the first in our History of Medicine series.

At the end of the first year, the journal is thriving. The journal has a 15-member editorial board, an editor, and managing and assistant managing editors. Except for some administrative assistance and the actual publishing costs, everything is done voluntarily. Our financing has three sources: a $350 publication fee for non-invited articles, donations from our readership (fully tax deductible), and generous financial support from MedStar Health System based in Columbia, Maryland, and the Union Memorial Hospital Department of Medicine in Baltimore.

If you have not read the journal, please sign up. It is a remarkable technology. Depending on the timing, it is not difficult to publish in final form articles that were submitted not more than 3 months prior. This has happened on a number of occasions so far this year.

We believe that the future of JCHIMP lies in its constituency in the community hospital network, that is, the Association of Program Directors and the voluntary subgroup, Community Hospital Education and Research Network (www.CHERNINFO.net). We foresee the journal as serving as an enduring publishing arm of CHERN.

We are very pleased with our peer reviewers. In the first four issues, 79 volunteers have completed reviews and are listed in Table 1. It is the first time some have been peer reviewers. 82% of those we requested have completed their reviews in the allotted 3 weeks. All submitted articles, including those invited, are peer reviewed by two or three peer reviewers. All peer reviewers are blinded to the origins of the articles. The journal editorial staff, with the help of David Solomon, is planning a faculty development workshop on peer reviewing in 2012.

**Table 1:** JCHIMP reviewers

Carlos Acuna	Steven Gambert	Phil Panzarella
Majd AI Ghatrif	Eric Goldberg	Anne Pereira
Chuck Albrecht	Mohammadali Habibi	Khalid Qazi
Donna Astiz	Mitra Hashemi	Ashish Rana
William Barnhart	Youssef Hokayem	Paul Sack
J Bergquist	Noah Hoskins	Fardad Sarabchi
Paul Bernstein	Rong Hu	Pamela Schroeder
Wayne Campbell	Danjela Jelovac	Andrew Schuldenfrei
Harjit Chahal	Yan Ji	Nawar Shara
lssam Cheikh	Ning Ji	Mansur Shomali
Malek Cheikh	Joel Katz	Peter Jeffery Sloane
Meegan Chestnut	Christopher Kearney	Cynthia Smith
Ehshan Chitsaz	Bassem Khalil	David Gary Smith
Chester Choi	Ramesh Khurana	Linda Thomas
Dobbin Chow	Robert Kornberg	Radika Vij
John Cmar	Mahesh Krishnamurthy	David Weisman
Melissa Delong	Claudia Kroker	Neil Weissman
Stephanie Detterline	Sapna Patel Kuehl	Wouter Wieling
Tracey Doering	Henry Meilman	Ashley Wietsma
Norman Dubin	Marita Mike	Donna Williams
Norman Dy	Himu Minn	Frederick Williams
Emmanuel Elueze	Suneet Mittal	Richard Williams
Fer Eren	Mahsa Mohebtash	Eskandar Yazaji
Robert Ferguson	Marc Mugmon	
Paul Foster	Daryn Norwood	
Dana Frank	Zahra Pakbaz	
Ethan Fried	Venkataraman Palabindala	

What we have accomplished to date? More than 2,500 ‘unique individuals’ have read the journal from 88 countries as of December 1, 2011. We have established our manuscript and article style – brief, succinct, and with a teaching message. Trainees are included at all levels in the organization, including readers and reviewers.

Where do we go from here? Our spring issue will have a diabetes treatment theme and will also include the first in our History of Medicine series. We plan to apply for ‘indexing,’ once we have the requisite number of articles published. We continue to recruit more reviewers and get them to engage in the program. As we become more widely known, we hope to have a broader scope; although, 88 countries is a good start.

This issue contains 11 manuscripts, including this one (1, 2) and a perspective piece on why case reports are important. Our first medical imaging report is from **David Widlus** (3). Two electrocardiogram are offered showing Osborne waves (4), and an upsettingly rapid atrial flutter with 1:1 conduction by **Marc Mugmon** (5). A perspective on the treatment of hyperkalemia and literature review are included (6). Four case reports by resident first authors are presented: hereditary renal carcinoma (7), Moxifloxacin–warfarin interaction (8), fatal meningococcemia (9), and ‘my appendectomy’ (10). The latter addresses what happens when a chief resident becomes a patient in his own institution. What could be a companion piece to this is a survey regarding how residents feel when receiving care for their personal health needs in their own institutions (11).

We will continue to publish quarterly. There is no limit to the number of manuscripts in each issue in our paperless journal. No limit except for the capacity of our volunteers. It is a lot of work, but it is also a lot of fun. We believe that what we are doing has an educational impact at many levels. I must confess that I love working with the students, residents, and faculty and hope that we are making a difference, raising their scholarly horizons. We believe that we are having an educational impact. We try to do it with a trace of wit because we believe that humor makes teaching fun.

*Robert P. Ferguson, MD* Editor

## References

[1] Ferguson R (2012). Anniversary issue. J Comm Hos Intern Med Perspect.

[2] Ferguson R (2012). The value of reporting cases. J Comm Hos Intern Med Perspect.

[3] Widlus D (2012). CT scan for suspect acute appendicitis. J Comm Hos Intern Med Perspect.

[4] Serafi S, Vliek C, Taremi M (2012). Osborne waves in a hypothermic patient. J Comm Hos Intern Med Perspect.

[5] Mugmon M (2012). Atrial flutter with 1: 1 conduction. J Comm Hos Intern Med Perspect.

[6] Mushiyakh Y, Dangaria H, Shahbaz Q, Noorjahan A, Pannone J, Tompkins D (2012). Treatment and pathogenesis of acute hyperkalemia. J Comm Hos Intern Med Perspect.

[7] Jadidi N, Mustafa S, Rodriguez R, Faraj S (2012). A case of hereditary papillary renal cell carcinoma. J Comm Hos Intern Med Perspect.

[8] Ji Y, Hokayem Y (2012). Moxifloxacin-coumadin interaction. J Comm Hos Intern Med Perspect.

[9] Tabacco J, Suniega E, Sarabchi F, Mitsani D (2012). Fatal meningococcemia. J Comm Hos Intern Med Perspect.

[10] Chiekh M (2012). My appendectomy. J Comm Hos Intern Med Perspect.

[11] Venkataraman P (2012). Personal health care of internal medicine residents. J Comm Hos Intern Med Perspect.

